# Machine learning model based on survey assessment of sleep quality in chronic obstructive pulmonary disease patients

**DOI:** 10.1371/journal.pone.0324480

**Published:** 2025-05-21

**Authors:** Miraç Öz, Banu Eriş Gülbay, Barış Bulut, Elif Akıncı Aydınlı, Aslıhan Gürün Kaya, Öznur Yıldız, Turan Acıcan, Sevgi Saryal

**Affiliations:** Department of Chest Diseases, Faculty of Medicine, Ankara University, Ankara, Türkiye; Charité - Universitätsmedizin Berlin, GERMANY

## Abstract

**Purpose:**

The aim is to develop a learning model based on clinical and survey data to assess sleep quality and identify determining factors affecting sleep quality in chronic obstructive pulmonary disease (COPD) patients.

**Methods:**

The Pittsburgh Sleep Quality Index (PSQI) was administered to stable COPD patients to assess sleep quality. Patients were categorized into two groups: good sleep quality and poor sleep quality. Parameters for the best model were selected from a total of 61 clinical and laboratory parameters using recursive feature elimination (RFE) and the Bayesian Information Criterion (BIC). A logistic regression (LR) model was created. The model was evaluated using nested cross-validation with 5 inner and 5 outer folds, and this process was repeated with 1000 bootstrap iterations. Results were obtained with a 95% CI.

**Results:**

The mean age of the 132 patients was 66.68 ± 8.16 years, with a predominance of males (117, or 88.6%). Of the 132 patients, 68 were in the poor sleep quality group. In this group, the prevalence of dyspnea, snoring, witnessed apneas, and excessive daytime sleepiness (EDS) was higher. The parameters included in the model and occurrence rates in the poor sleep quality group are as follows: annual exacerbation and hospitalization (71.9%), presence of EDS (35.9%), cough (64.1%), active smoking (95.4%), short-acting beta agonist (SABA) requirement (59.4%), pH level, and coronary artery disease (CAD) (20.3%). In our final model, the test set demonstrated a sensitivity, specificity, accuracy, and AUC of 70.21%, 71.76%, 70.99%, and 0.757, respectively.

**Conclusion:**

Our machine learning model, developed using clinical data of COPD patients, can predict their sleep quality. We found that high annual exacerbation and hospitalization rates, the presence of EDS and cough symptoms, active smoking, and regular use of SABA as well as high pH levels, negatively affect sleep quality. Conversely, the presence of CAD under treatment in patients positively affects sleep quality.

## Introduction

Chronic obstructive pulmonary disease (COPD) is a heterogeneous lung disease that is characterized by chronic respiratory symptoms (such as dyspnea, cough, sputum production, and exacerbations) caused by irreversible, usually worsening obstruction of airflow in the airways (bronchitis, bronchiolitis) and abnormalities in the air spaces (emphysema) [1].

Comorbidities in COPD impact symptoms, quality of life, complications, treatment, economic burden, and mortality [1]. One of the common comorbid conditions in COPD patients is sleep problems. Approximately 40% of COPD patients experience sleep-related problems. Insomnia, non-restorative sleep, and excessive daytime sleepiness (EDS) are common symptoms in patients with COPD. Polysomnographic studies have demonstrated that COPD patients frequently struggle with initiating or maintaining sleep and experience varying degrees of impaired sleep efficiency. In addition, they exhibited shorter sleep durations, reduced Rapid Eye Movement (REM) sleep, decreased Non-Rapid Eye Movement (NREM) Stage 3 sleep, frequent shifts between sleep stages, and micro-arousals, indicating significant changes in sleep structure [2,3]. In patients with COPD, sleep disruptions can occur at the onset of sleep and can also be related to the onset of COPD symptoms during late night or early morning hours. Poor sleep quality is thought to lead to increased COPD symptoms and mortality. While the specific effect of chronic sleep problems on pulmonary function tests in patients with COPD is not well understood, research has indicated that it may contribute to a slight, yet potentially significant, decrease in forced vital capacity (FVC) and forced expiratory volume in one second (FEV_1_) [4].

The COPD Assessment Test (CAT) score and modified Medical Research Council (mMRC) dyspnea scale may indicate a link between COPD severity and subjective sleep quality. The COPD stage is clinically significant. Studies have demonstrated that in patients with recurrent COPD exacerbations, sleep quality is poorer due to symptoms such as reduced sleep length, increased dyspnea-related disturbances, and early awakening [2,3,5]. Furthermore, the presence of other accompanying comorbidities and the use of medical treatments can also impact sleep quality.

Prior research has employed machine learning and deep learning algorithms to predict sleep quality in COPD patients. These studies have commonly incorporated factors such as time spent in bed and sleep latency, based on data collected from wearable devices [6].

CAT includes a question that assesses whether COPD patients sleep soundly, but we have not found a machine learning model based on freely accessible and frequently used demographic and verified survey data. Our project aims to develop a learning model to estimate COPD patients’ sleep efficiency using clinical and survey data without polysomnographic or wearable device data [7].

## Materials and methods

Our study was conducted prospectively between January 01, 2023, and January 01, 2024, including stable COPD patients who presented to the Chest Diseases outpatient clinic, with the approval of the Ankara University Faculty of Medicine Ethics Committee dated August 01, 2022 (approval number: İ07-397-22). The inclusion criteria were age > 40 years old, willingness to participate in the study, diagnosis of COPD, stable COPD status, compliance with spirometry, absence of any malignancy diagnosis, absence of known psychiatric illness or medication for psychiatric conditions, and absence of known sleep-disordered breathing diagnosis or use of Positive Airway Pressure (PAP) therapy.

Patients who were accepted for the study provided informed consent. We recorded demographics, smoking habits, comorbidities, duration of COPD diagnosis, annual COPD exacerbations, hospitalizations, and both medical and non-medical treatments, such as long-term oxygen therapy (LTOT). Symptoms were assessed. For stable COPD patients, spirometry, arterial blood gas, complete blood count, and glomerular filtration rate were documented within six months. Patients’ COPD stages and sleep quality were assessed using the following questionnaires.

### COPD assessment test (CAT)

All participants completed the validated Turkish version of the CAT to assess COPD symptoms and associated health status [8,9]. The 8-question CAT includes questions on coughing, sputum production, chest tightness, activity limitations, sleep quality, energy level, dyspnea, and housework. Based on patient symptoms, each question is scored from 0 (no impact) to 5 (very severe). The total score ranges from 0 to 40. Higher ratings indicate more severe symptoms and a greater impact of COPD on the patient’s health [9].

### The modified medical research council (mMRC)

The Modified Medical Research Council (mMRC) scale measures the severity of dyspnea based on the physical activities that trigger it. Scores range from 0 (breathless only with challenging physical activity) to 4 (too breathless to leave the home or dress and/or undress) [10].

### The epworth sleepiness scale (ESS)

ESS measures EDS by asking about the likelihood of falling asleep in various situations (0 = none, 3 = high likelihood). The test consists of eight Turkish-validated questions [11]. Scores range from 0–24, with a score of 10 or higher indicating EDS [12].

### The pittsburgh sleep quality ındex (PSQI)

PSQI is a questionnaire developed worldwide to assess subjective sleep quality. It evaluates seven components of sleep quality and has been validated in Turkish for use in healthy individuals and various patient groups [13]. These components include patients’ subjective sleep quality, sleep latency, sleep duration, sleep efficiency, presence of sleep disturbances, use of sleep medication, and the impact of current sleep problems on patients’ daytime functioning. The total score ranges from 0 to 21, with a score greater than 5 indicating poor sleep quality [14].

### STOP-BANG

The STOP-BANG scoring system consists of eight questions, all answered in a yes-no format, with each yes answer scored as 1 point. Each question is named by combining the initial letters of the key terms in its content: snoring, excessive daytime sleepiness or feeling sleepy, observed cessation of breathing during sleep, high blood pressure, body mass index > 35 kg/m^2^, age > 50, neck circumference > 40 cm, male gender [15,16]. The scoring system has been validated in Turkish [17].

### The hospital anxiety and depression scale (HADS)

HADS, validated in Turkish, was used to determine the presence of depression and anxiety in patients [18–20]. HADS consists of 14 items, with each subscale containing 7 items, and a total score ranging from 0 to 21. Scores of 7 and below on each subscale are considered normal, while scores of 11 or higher indicate the possible presence of a mood disorder. High scores on each subscale indicate the presence of severe anxiety and/or depression [19].

### Machine learning model

Since no single parameter in the study consistently predicted sleep quality accurately, the parameters were used to develop a machine learning model using a logistic regression (LR) algorithm. The LR algorithm was chosen not only for its ability to predict sleep quality but also for its capacity to analyze the effect of model parameters on the outcome.

From the 61 parameters in the study, candidate parameters were selected using the recursive feature elimination (RFE) method. To avoid overfitting due to the low number of cases, parameters selected via RFE were further evaluated using the Bayesian Information Criterion (BIC) method, which penalizes more complex models, to achieve the best model. Before advancing to further stages in the LR model, the risk of multicollinearity among the parameters was assessed using the Variance Inflation Factor (VIF), and it was expected that the VIF value for all parameters would be less than 3.0. Additionally, the fit of the model’s predictions to the observed results was evaluated using the Hosmer-Lemeshow goodness-of-fit test and a calibration plot.

The success of the model was evaluated using nested cross-validation (nested-CV). To achieve this, the dataset was divided into five inner and five outer folds. Hyperparameter optimization was performed using the inner loops, while sensitivity, specificity, accuracy, and AUC metrics were calculated using the outer loops. This process was repeated with 1000 iterations of bootstrap resampling, and cumulative results were reported as the mean (95% CI) for training and validation sets.

The effectiveness of the model in predicting sleep quality was compared to the CAT score using ROC analysis with the DeLong method [21]. Additionally, the model’s net benefit at different threshold values was evaluated using decision curve analysis (DCA). This evaluation was visualized for both low and overall threshold probabilities and compared to the CAT score. To enhance the model’s user-friendliness, a nomogram was developed.

### Statistical analysis

A Shapiro-Wilk test was used to determine whether the research parameters were normally distributed. Parameter results were compared using the t-test for normally distributed parameters and the Mann-Whitney U test for non-normally distributed parameters. Chi-square or Fisher’s exact tests were used to compare nominal data. IBM SPSS Statistics 28.0 was used for descriptive statistics, t-tests, and Mann-Whitney U tests, while MedCalc 19.2.6 was used for ROC analysis. RFE, BIC analysis, the Hosmer-Lemeshow test, Nested-CV, outcome metrics, and DCA visualization were performed in Python 3.9. The nomogram was generated using R statistical computing.

Power analysis of the study was conducted using G*Power 3.1.9.2

## Results

**Fig 1:**
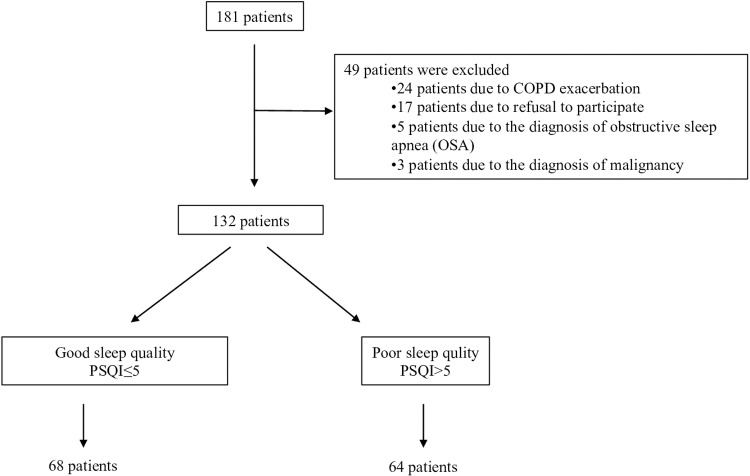
Flowchart: Distribution of patients according to PSQI results.

The study included 132 patients diagnosed with stable COPD who consented to participate. These patients were categorized based on their sleep quality as assessed by the PSQI. The distribution was as follows: 68 patients (51.5%) were classified as having good sleep quality (PSQI ≤ 5), while 64 patients (48.5%) exhibited poor sleep quality (PSQI > 5), as depicted in Fig. 1.

The mean age of the 132 patients enrolled was 66.68 ± 8.16 years, with a predominance of male participants (117 or 88.6%). Statistical analysis revealed no significant differences in age or gender between groups stratified by sleep quality, indicating that these variables did not influence the grouping. Similarly, the BMI distribution remained consistent across both groups.

Dyspnea emerged as the most reported symptom among all participants and was more prevalent in those with poor sleep quality (p = 0.033). Additionally, symptoms indicative of obstructive sleep apnea (OSA)—such as snoring, witnessed apneas, and EDS—were significantly more frequent in the poor sleep quality group (p values = 0.023, 0.026, and 0.017, respectively).

There were no significant differences in the duration of COPD diagnosis or the annual number of COPD exacerbations between the groups. Most patients in both groups were categorized into COPD Group E, suggesting a severe stage of the disease with frequent exacerbations and significant airflow limitations.

The prevalence of comorbidities, particularly congestive heart failure (CHF), was notably higher in the poor sleep quality group (p = 0.036), suggesting a link between sleep quality and cardiovascular health in COPD patients. Regarding COPD-specific treatments, the use of inhaled corticosteroids (ICS), long-term oxygen therapy (LTOT), and nebulizers was significantly higher among those with poor sleep quality (p values = 0.027, 0.005, and 0.047, respectively), indicating a potentially greater disease burden and increased healthcare utilization in this group (Table 1).

**Table 1:** Demographic and Clinical Characteristics of Patients.

	Good sleep quality (n: 68)	Poor sleep quality (n:64)	p value
**Gender, M, n (%)**	62 (91.2)	55 (85.9)	0.343
**Age (year) mean ± SD**	67 ± 7.79	66.34 ± 8.59	0.646
**BMI (kg/m** ^**2**^ **), mean ± SD**	26.24 ± 4.89	27.25 ± 5.51	0.269
**Smoking history**			
**Never, n (%)**	2 (2.9)	3 (4.7)	0.599
**Current, n (%)**	24 (35.3)	28 (43.8)	0.320
**Previous, n (%)**	42 (61.8)	33 (51.6)	0.237
**Alcohol**			
**Current, n (%)**	12 (17.6)	8 (12.5)	0.410
**Never, n (%)**	56 (82.4)	56 (87.5)	0.410
**Symptoms, n (%)**			
**Snoring**	28 (41.2)	39 (60.9)	**0.023**
**Witnessed apnea**	11 (16.2)	21 (32.8)	**0.026**
**EDS**	12 (17.6)	23 (35.9)	**0.017**
**Headache**	13 (19.1)	26 (40.6)	**0.007**
**Night sweats**	16 (23.5)	21 (32.8)	0.239
**Dry mouth**	31 (45.6)	41 (64.1)	**0.033**
**Nocturia**	33 (48.5)	34 (53.1)	0.601
**Tachycardia**	9 (13.2)	15 (23.4)	0.131
**Cough**	29 (42.6)	41 (64.1)	**0.014**
**Dyspnea**	48 (70.6)	55 (85.9)	**0.033**
**Sputum**	36 (52.9)	40 (62.5)	0.267
**Chest pain**	8 (11.8)	16 (25)	**0.049**
**COPD evaluation**			
**Group A, n (%)**	17 (25)	6 (9.4)	**0.018**
**Group B, n (%)**	23 (33.8)	19 (29.7)	0.613
**Group E, n (%)**	28 (41.1)	39 (60.9)	**0.023**
**Duration of illness, year, mean ± SD**	10.25 ± 7.11	10 [5-15.75]	0.468
**Annual exacerbation rate, median [IQR]**	0 [0-1]	1 [1–1]	0,670
**Annual exacerbation rate ≥1, n (%)**	27 (39.7)	46 (71.9)	**< 0.001**
**Annual hospitalization rate, median [IQR]**	0 [0-1]	1 [0-1]	**0.002**
**Comorbidities, n (%)**			
**GERD**	13 (19.1)	15 (23.4)	0.544
**DM**	11 (16.2)	12 (18.8)	0.697
**HT**	38 (55.9)	28 (43.8)	0.164
**CAD**	23 (33.8)	13 (20.3)	0.082
**BPH**	20 (29.4)	22 (34.4)	0.541
**CKD**	5 (7.4)	7 (10.9)	0.474
**CHF**	0	4 (6.3)	**0.036**
**Treatment, n (%)**			
**LABA**	49 (72.1)	55 (85.9)	0.051
**LAMA**	60 (88.2)	57 (89.1)	0.881
**ICS**	41 (60.3)	50 (78.1)	**0.027**
**SABA**	31 (45.6)	38 (59.4)	0.113
**Theophylline**	11 (16.2)	15 (23.4)	0.294
**Diuretic**	10 (14.7)	16 (25)	0.137
**MDI**	25 (36.8)	30 (46.9)	0.239
**LTOT**	16 (23.5)	30 (46.9)	**0.005**
**Use of Nebulizer**	13 (19.1)	22 (34.4)	**0.047**

M: Male, n: Number of patients, SD: Standard deviation, BMI: Body mass index, EDS: Excessive daytime sleepiness, COPD: Chronic obstructive pulmonary disease, IQR: Interquartile range, GERD: Gastroesophageal reflux disease, DM: Diabetes mellitus, HT: Hypertension, CAD: Coronary artery disease, BPH: Benign prostatic hyperplasia, CKD: Chronic kidney disease, CHF: Congestive heart failure, LABA: Long-acting beta-agonist, LAMA: Long-acting antimuscarinic, ICS: Inhaled corticosteroid, SABA: Short-acting beta-agonist, MDI: Metered-dose inhaler, LTOT: Long-term oxygen therapy

The analysis of arterial blood gas revealed a higher partial pressure of carbon dioxide (pCO_2_) in patients classified with poor sleep quality (p = 0.034). However, the partial pressure of oxygen (pO_2_) did not significantly differ between the two groups.

Regarding spirometry measures, FEV_1_ as a percentage of the predicted value was significantly lower in the poor sleep quality group (p = 0.037). This finding correlates with the observed higher symptom burden and potentially more advanced disease state in this group (Table 2).

**Table 2:** Comparison of Groups in Terms of Arterial Blood Gas, Laboratory, and Spirometry Results.

	Good sleep quality (n: 68)	Poor sleep quality (n:64)	p value
**Arterial blood gas analysis**			
**pH, median [IQR]**	7.41 [7.39-7.44]	7.41 [7.39-7.43]	0.642
**pCO**_**2**_ **, (mmHg), median [IQR]**	38.05[34.52-41.95]	41 [35.17-46.55]	**0.034**
**pO**_**2**_ **, (mmHg), mean±SD**	63.79 ± 12.60	62.42 ± 14.81	0.566
**SaO**_**2**_ **(%), median [IQR]**	93 [89.27-94.97]	92 [85.62-95]	0.411
**HCO**_**3**_ **, (mEq/L), median [IQR]**	25 [23.22-26.17]	25.4 [23.85-28.22]	0.075
**Spirometry**			
**FVC % predicted, mean±SD**	79.48 ± 17.79	73.34 ± 20.79	0.070
**FEV**_**1**_ **% predicted, median [IQR]**	55.50[42.00-71.00]	46.5 [37-62.75]	**0.037**
**FEV**_**1**_ **/FVC %, mean±SD**	56.08 ± 11.30	54.96 ± 13.29	0.604
**FEF25%-75%, % predicted, median [IQR]**	19 [13.75-38.00]	17.5 [12-29.5]	0.444
**Laboratory**			
**Hb, (gr/dL), median [IQR]**	14.65 [13.22-15.87]	14 [12.72-15.37]	0.072
**Hct, (%), median [IQR]**	44.85 [41.40-48.50]	42.60 [38.77-47.65]	0.061
**GFR, (mL/dk/1.73m** ^**2**^ **, median [IQR]**	85.50 [74-91]	87 [71.25-96.75]	0.489

*n: number of patients, IQR: interquartile range, pCO2: partial pressure of carbon dioxide, mmHg: millimeters of mercury, pO*_*2*_*: partial pressure of oxygen, SD: standard deviation, SaO*_*2*_*: oxygen saturation, HCO3: bicarbonate, FVC: forced vital capacity, FEV1: forced expiratory volume in one second, FEF %25-%75: forced expiratory flow between 25% and 75%, Hb:* hemoglobin*, gr/dL: grams per deciliter, Hct: hematocrit, GFR: glomerular filtration rate, mL/min: milliliters per minute*

The study demonstrated a significant association between poor sleep quality, increased daytime sleepiness, and a higher risk for OSA. The ESS scores were notably higher in the poor sleep quality group (p = 0.003). Similarly, STOP-BANG scores were significantly greater in this group (p = 0.019).

Further analysis revealed that COPD patients with good sleep quality reported lower severity in COPD symptoms and associated health status impairment, as measured by the CAT score and the mMRC dyspnea scale. Both CAT and mMRC scores were significantly lower in the good sleep quality group (p < 0.001) (Table 3).

**Table 3:** Comparison of dyspnea severity, health status, Hospital Anxiety and Depression Scale Results between groups.

	Good sleep quality (n: 68)	Poor sleep quality (n:64)	p value
**mMRC, median [IQR]**	1[1–2]	2[1–3]	**< 0.001**
**CAT score, median [IQR]**	11 [6.25–15.75]	20 [12.5–26]	**< 0.001**
**ESS, median [IQR]**	3 [2–6]	5.5 [2.25-9]	**0.003**
**STOP-BANG score median [IQR]**	3 [2.25–4]	4 [3–5]	**0.019**
**Anxiety score, median [IQR]**	3.5 [1–6.75]	7 [3–10.75]	**< 0.001**
**Anxiety point, n (%)**			
**Normal**	54 (79.4)	40 (62.59	**0.032**
**Borderline**	11 (16.2)	8 (12.5)	0.551
**Anxiety**	3 (4.4)	16 (25)	**0.001**
**Depression score, median [IQR]**	5 [2–8]	7.5 [4–10]	**0.001**
**Depression point, n (%)**			
**Normal**	48 (70.6)	32 (50)	**0.015**
**Borderline**	15 (22.1)	22 (34.4)	0.117
**Depression**	5 (7.4)	10 (15.6)	0.137

*n: patient count, IQR: interquartile range, mMRC: modified Medical Research Council, CAT: COPD assessment test, ESS: Epworth Sleepiness Scale, STOP-BANG: Snoring, Tiredness, Observed apnea, Pressure- BMI, Age, Neck circumference, Gender*

Depression and anxiety scores were significantly lower in the good sleep quality group (p = 0.001, p < 0.001), respectively (Table 3).

In subgroup analyses focusing on smoking status and the use of ICS, significant differences emerged in annual hospital admissions among patients categorized with poor sleep quality. Specifically, these patients had more hospital admissions annually compared to those with good sleep quality (p = 0.010). In contrast, no significant differences were observed in patients using SABA regarding hospital admissions (p = 0.052). However, among SABA users, the annual rate of exacerbations was significantly greater in the poor sleep quality group (p = 0.006).

### The logistic regression model

**Fig 2:**
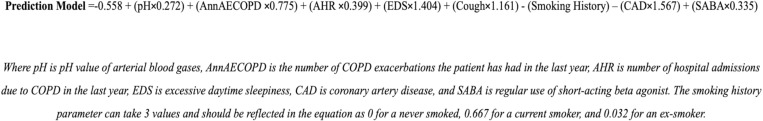
Final model of study.

Initially, all 61 parameters in the dataset were evaluated using RFE, identifying the most valuable 18 parameters for predicting the binary outcome. To avoid overfitting and multicollinearity, these 18 parameters were not included in the model directly. Instead, models containing possible combinations of 8–11 parameters from this set were generated and compared using the BIC score. The model with the lowest BIC score, containing 8 parameters, was selected as the final model for the study (Fig 2).

**Fig 3:**
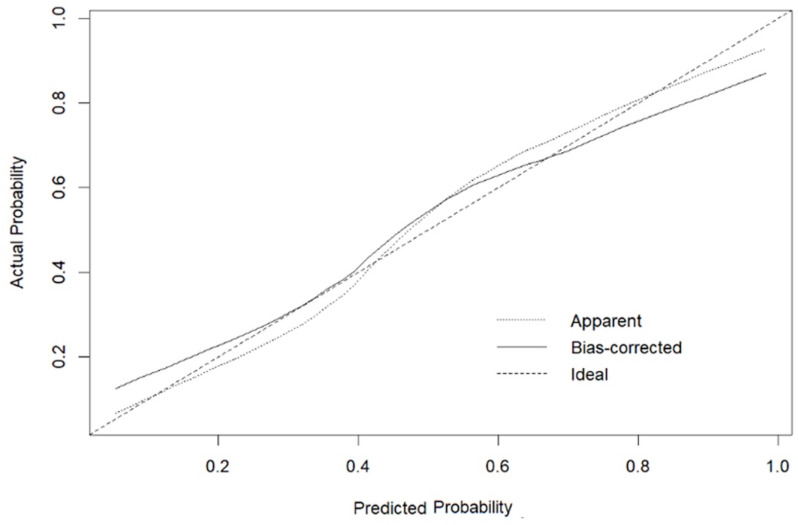
Calibration Plot of the Logistic Regression Model.

The Hosmer-Lemeshow goodness-of-fit test indicates that the model’s predictions fit well with the observed outcomes in the dataset (p = 0.473). When examining the calibration plot, it is evident that the model does not exhibit issues of overestimation or underestimation (Fig 3).

The odds ratios of the model parameters and the metrics obtained from the training and test sets are provided in Table 4. Statistically significant variables in this model, determined by RFE and BIC methods, were Attack Number, EDS, Cough, and CAD.

**Table 4:** Study model: Multivariate Analysis of Parameters and Performance Metrics.

Multivariate analysis results of the Study Model Parameters			Performance Metrics of the Study Model							
**Parameter**	**p**	**Odds ratio** **(95%CI)**	Training set*				Test set*			
			**Sens%** **(95%CI)**	**Spec%** **(95%CI)**	**Acc%** **(95%CI)**	**AUC** **(95%CI)**	**Sens%** **(95%CI)**	**Spec%** **(95%CI)**	**Acc%** **(95%CI)**	**AUC** **(95%CI)**
pH**	0.212	1.309 (0.856-2.014)	77.27 (77.10–77.44)	74.02 (73.82–74.,23)	75.62 (75.47% -75.76%)	0.826 (0.825 -0827)	70.21 (69.09–71.33)	71.76 (70.64 –72.88)	70.99 (70.19–71.78)	0.757 (0.74–0.767)
Attack**	0.017	2.171 (1.151-4.094)								
Hospital**	0.136	1.491 (0.882-2.519)								
EDS	0.007	4.072 (1.468-11.296)								
Cough	0.009	3.194 (1.322-7.719)								
Smoking (1)	0.725	0.513 (0.095-2.766)								
Smoking (2)	0.438	0.968 (0.314-2.983)								
CAD	0.004	0.209 (0.072-0.608)								
SABA	0.441	1.398 (0.592-3.302)								

* Results of 1000-bootstrap iterations of 5-fold inner and 5-fold outer loop nested cross validation, Sens: Sensitivity, Spec: Specificity, Acc: Accuracy, AUC: Area under curve

** Results of standardized values *of the parameter*

*pH: pH value in arterial blood gas, attack: Number of COPD attacks the patient has had in the last year, hospital: Hospitalization due to AE-COPD/year, EDS: excessive daytime sleepiness, Smoking (1):* current *smoker, Smoking (2): ex-smoker CAD: coronary artery disease, SABA: short acting beta agonist*

The model’s performance metrics indicate that there is no overfitting issue (Table 4). The AUC value in the test set was calculated as 0.757. In this model, an increased smoking score and having CAD were found to increase the likelihood of the patient having better sleep quality. An increase in quantitative parameters, including pH, raises the probability of the patient being labeled as having poor sleep quality when compared other qualitative parameters.

**Fig 4:**
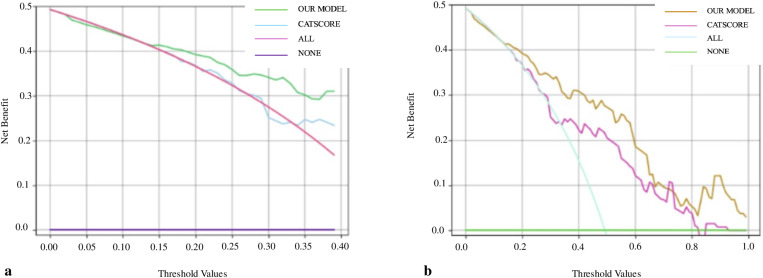
a. Comparison of CAT score and our model in predicting sleep quality at low threshold values. b. Comparison of CAT score and our model in predicting sleep quality at high threshold values.

Using the CAT score to predict sleep quality, which evaluates the health status of COPD in the study group, and applying the same cross-validation method, the sensitivity, specificity, accuracy, and AUC in the test set were found to be 69.14%, 73.92%, 71.55%, and 0.739, respectively. Comparing our model to the CAT score using the DeLong method revealed no significant difference (p = 0.225). The effectiveness of the model at different threshold probabilities was evaluated using DCA. At low threshold values, the CAT score does not provide a net benefit in predicting sleep quality, whereas our model presents a significant net benefit (Fig 4a). At high threshold values, the DCA curve of our model has a higher AUC value compared to the CAT score (Fig 4b).

**Fig 5:**
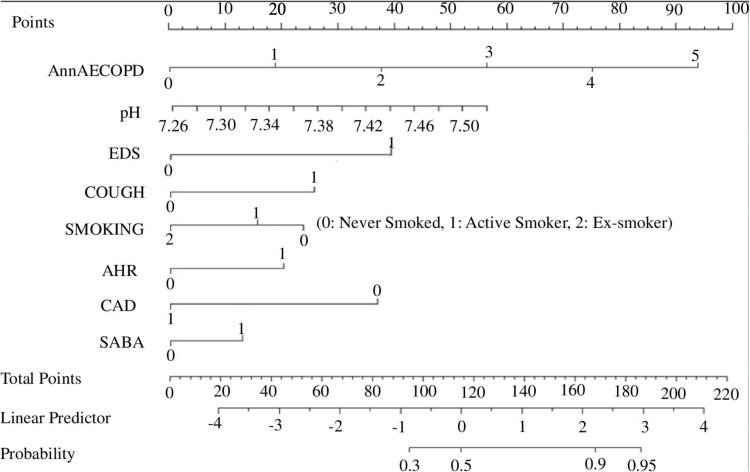
The prediction nomograms of risk factors for poor sleep quality in COPD (*AnnAECOPD: annual acute exacerbation chronic obstructive lung disease, EDS: excessive daytime sleepiness, AHR: annual hospitalisation rate, CAD: coronary artery disease, SABA: short acting beta agonist).*

A nomogram created for the ease and quick utilization of the model is provided in Fig 5.

### Model performance and odds ratios

The logistic regression model’s performance was favorable, with an area under the curve (AUC) for the test set calculated at 0.757, indicating good discriminative ability to differentiate between patients with good and poor sleep quality. The odds ratios for the significant model parameters—Attack Number, EDS, Cough, and CAD—highlighted their respective impacts on sleep quality outcomes.

## Discussion

Assessing sleep quality in patients with COPD is crucial for improving their overall health and patient care. The current literature indicates that various machine learning models have been developed to predict sleep quality based on data collected using wearable devices or polysomnography [6,22]. However, these methods may not be practical for individuals living in low-income areas or with limited access to technology, and the continuous use of wearable devices may be uncomfortable for patients. Therefore, there is a significant need for a machine learning model that can predict sleep quality using clinical data from COPD patients. In our model developed for this purpose, we found that a high number of annual exacerbations and hospitalizations, the presence of EDS and cough symptoms, a history of active smoking and regular SABA use, and high pH levels negatively impact sleep quality, whereas the presence of CAD under treatment in patients positively affects sleep quality.

In a study examining the sleep quality of 1,117 patients diagnosed with moderate to severe COPD, it was found that patients with poor sleep quality had a significantly higher number of annual exacerbations compared to those with good sleep quality (1.7 vs. 1.37) [5].

Another multicenter cohort study of 1,647 COPD participants reported that poor sleep quality, as measured by the PSQI, was a significant risk factor associated with both the number of exacerbations and hospitalizations [23]. Our study supports previous findings, indicating that sleep quality is linked to COPD exacerbations and hospitalizations, thereby contributing to the literature. Our results align with prior studies showing an increased risk of exacerbations in COPD patients with poor sleep quality [24,25]. One of these studies, despite considering socioeconomic status, had limitations such as a small sample size, outdated measurements for COPD severity, and limited consideration of comorbidities [23]. Shorofsky et al., in a multicenter Canadian cohort, found that after adjusting for demographic characteristics, COPD severity, comorbidities, and medication use, the time to first exacerbation was shorter in the group with poor sleep quality [24]. Conversely, a study on the use of azithromycin in frequent COPD exacerbations indicated that the unadjusted relationships between sleep quality and the number of exacerbations were largely related to comorbidities and medication use [5]. In conclusion, poor sleep quality in patients has been associated with adverse outcomes, such as exacerbations and hospitalizations [26].

Various symptoms determining sleep quality in COPD patients were included in our study model. The first among these is the presence of EDS, which is thought to result from factors such as a drop in oxygen saturation during sleep, frequent awakenings, and the sedative effect of CO_2_ retention on the central nervous system in advanced stages of COPD, depressive mood due to comorbid diseases, and possible side effects of medications used by patients [27]. It is known that EDS is present in 8% of COPD patients according to a study investigating sleep quality [28]. In contrast to this study, 26.5% of all patients in our study described EDS, with this rate being 35.9% in COPD patients with poor sleep quality. In our study, we should note that we determined the presence of EDS based on patients’ responses to a question similar to the STOP-BANG scoring system, rather than the ESS score. Additionally, the ESS score was statistically higher in COPD patients with poor sleep quality. Ultimately, in our model, we found that describing EDS was one of the parameters determining poor sleep quality among symptoms. The presence of OSA may also be a reason for EDS in COPD patients. In previous studies, COPD patients with poor sleep quality had higher STOP-BANG scores, and the cardinal symptoms of OSA (snoring, witnessed apnea, and EDS) were more frequently observed [29]. However, the STOP-BANG score was not directly included in our model, possibly due to the overlapping variables with other parameters.

The presence of cough is another symptom considered significant in our model for poor sleep quality. While 64.1% of COPD patients with poor sleep quality had a cough symptom, this rate was 42.6% in those with good sleep quality. We believe that cough complaints affect sleep quality by causing frequent awakenings, prolonging sleep onset, and shortening total sleep duration. Our study has limitations, as we only evaluated cough as “present or absent” without using a scale to assess its frequency and severity.

There seems to be more consistency among studies examining the relationship between smoking and sleep disturbance. Acute nicotine withdrawal during sleep and the resulting sympathetic activation are possible explanations for the adverse effect of smoking on sleep quality [29]. Similar to previous studies, our model found that sleep quality was better in patients who had quit smoking. Surprisingly, we found that never having smoked was one of the determinants of poor sleep quality in our model. We noted that there were only five patients in our study who had never smoked, and we did not conduct a separate statistical analysis for this small group. We believe that further research with a larger group of never-smoking COPD patients is necessary. Additionally, the low number of female patients, the lack of inquiry into patients’ educational status, and the absence of questioning smoking status in pack-years, along with other risk factors for COPD, are significant limitations of our study.

Inhaled treatments used in COPD management have also been suggested to impact sleep quality. A study showed that the use of anticholinergics, beta-agonists, or inhaled corticosteroids did not influence the likelihood of poor sleep quality in a COPD cohort [29]. Current studies present mixed results regarding the impact of inhaler treatments on sleep quality. Medications used by patients can particularly affect sleep quality due to their side effects. In our study, when we reviewed the bronchodilators used by patients, we found that regular SABA use negatively affected sleep quality among the variables in our model. We can predict that patients requiring regular SABA use have more pulmonary symptoms and exacerbations. Additionally, potential side effects of SABA use, such as tachycardia and tremor, may also negatively influence patients’ sleep quality.

One of the parameters in our nomogram that we found challenging to explain was how an alkaline pH value led to poor sleep quality in COPD patients. Normally, the body’s pH balance is within a very stable range (blood pH approximately 7.35–7.45). However, in COPD patients, especially in advanced stages, this balance can be disrupted due to respiratory failure, leading to unexpected pH changes. Alkaline pH, often resulting from hyperventilation during a dyspneic process or metabolic reasons such as intense diuretic-steroid use, can cause both irregular breathing patterns during sleep (e.g., Cheyne-Stokes respiration) and sleep fragmentation, and it can affect oxygen and CO_2_ transport, making it harder for oxygen to reach tissues. In alkalosis, hemoglobin’s affinity for oxygen increases, and oxygen delivery to tissues decreases. This can lead to more hypoxia, especially during sleep. Alkaline pH can also reduce the effectiveness of respiratory muscles, increasing respiratory distress and negatively affecting sleep quality [30].

A study observed a higher prevalence of comorbid cardiac diseases, including myocardial infarction, peripheral artery disease, and hypertension, in patients with moderate to severe COPD. Other cardiovascular diseases, such as heart failure and arrhythmia, were also found to be higher in the moderate to severe COPD group compared to other groups, although not statistically significant [29]. In our study, only four patients had a diagnosis of heart failure, all of whom had poor sleep quality. Due to the small number of cases, this variable was not included in statistical comparisons. Another study noted an independent correlation between decreased lung function and increased risk of heart failure, CAD, and atrial fibrillation, in addition to showing an increased prevalence of insomnia in patients with cardiovascular diseases [31–33]. Taken together, it is possible that these comorbidities partly mediate sleep disturbances in COPD patients. Contrary to these studies, we found that the presence of CAD in COPD patients positively affected sleep quality. It is thought that COPD patients with CAD might have basic sleep problems due to serious complications in both the respiratory and cardiovascular systems. However, particularly effective interventions for CAD treatment (e.g., statin therapy, angioplasty, coronary bypass surgery) primarily improve the patient’s overall cardiovascular health. Thus, effective CAD treatment might enhance sleep quality in COPD patients by providing more efficient oxygenation throughout the night and reducing respiratory distress during sleep. Some medications used during heart disease treatment (e.g., beta-blockers) can also improve sleep quality by regulating heart rhythm during sleep. Additionally, CAD patients are typically encouraged by healthcare teams to make lifestyle changes, such as quitting smoking, exercising regularly, and eating healthily, which can also contribute to improved sleep quality. Meta-analyses in the literature indicate that rehabilitation programs following effective CAD treatment improve patients’ quality of life [34]. Our study did not include patients presenting with acute cardiac pathology, and only the known CAD presence was queried, without a detailed investigation of CAD.

Although the CAT score was high in COPD patients with poor sleep quality, the semi-quantitative and subjective results of this survey were not included in our model variables. Furthermore, our model’s DCA test result, compared with the CAT score’s DCA test result (Figs 4a, 4b), demonstrated that our model performed better in predicting sleep quality in COPD patients over a broad threshold range. This means that our model is more successful in predicting sleep quality in both low- and high-risk COPD patients compared to the CAT, a subjective test that includes a question assessing sleep quality. Ultimately, the DCA test indicated that the CAT score did not provide a net benefit in low-risk COPD patients for poor sleep quality.

In a study examining mental health and sleep quality in COPD, involving 190 stable COPD patients, advanced age and male gender were among the determinants of poor sleep quality [35]. However, in our study, age, gender, and BMI were not significant parameters for predicting sleep quality.

Although not included in our model, we found that total anxiety and depression scores were significantly higher in COPD patients with poor sleep quality (p < 0.001, p = 0.001). This supports previous findings indicating an association between depression and sleep-disordered breathing [36]. Additionally, past research has exhibited a relationship between sleep disorders and both depression and anxiety levels in COPD patients, consistent with our findings [35,37]. In our study, we demonstrated that the total depression score was statistically higher in COPD patients with ≥1 annual exacerbations, even without a significant difference in the total anxiety score (p = 0.099, p = 0.004). We did not assess patients’ educational and socioeconomic status; hence, a direct relationship between anxiety and depression scores and sleep quality could not be established, as these were not included in our model parameters.

The subjective assessment of patients’ sleep quality with a questionnaire and the lack of objective evaluation methods such as polysomnography, as well as the absence of questioning for restless leg syndrome, are limitations in our single-center study. Additionally, the low number of female patients and the lack of a healthy control group are other limitations.

## Conclusion

There is no model developed in the literature to predict poor sleep quality in COPD patients. In our study, the model we developed, which includes the number of annual exacerbations and hospitalizations, the presence of EDS and cough, smoking status, pH values, SABA use, and CAD presence, can be used to predict sleep quality in COPD patients.
